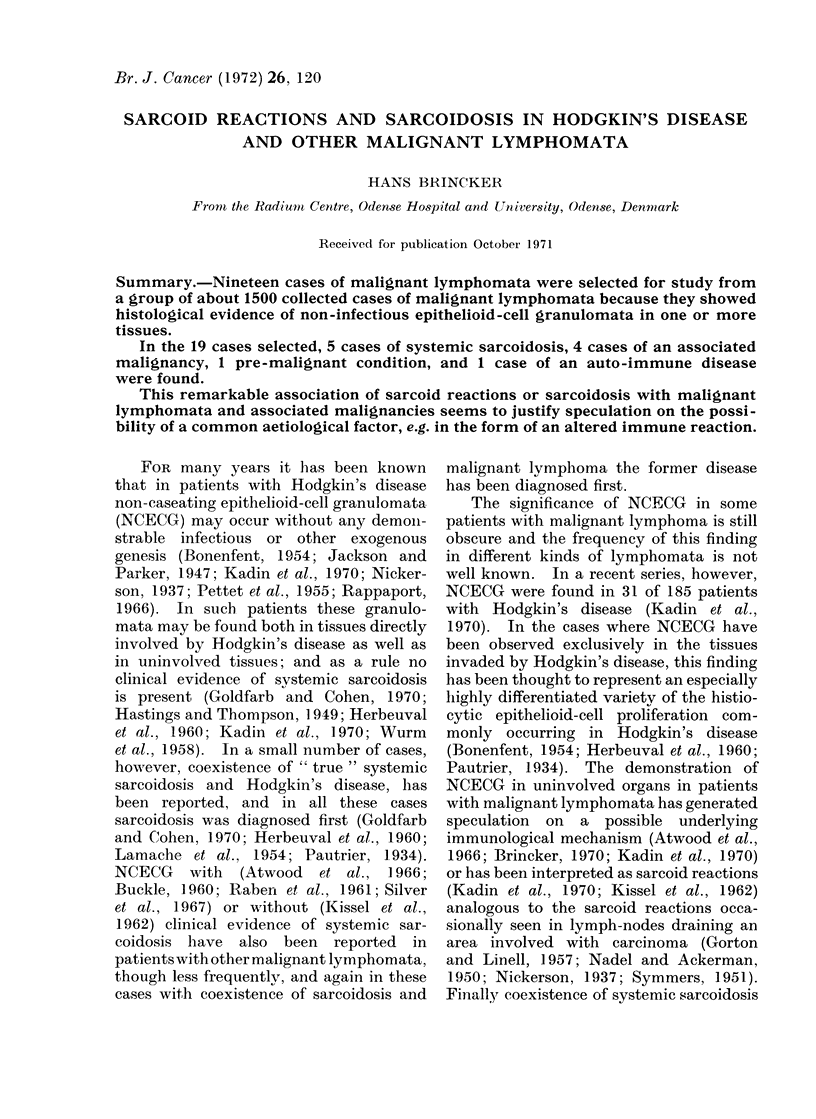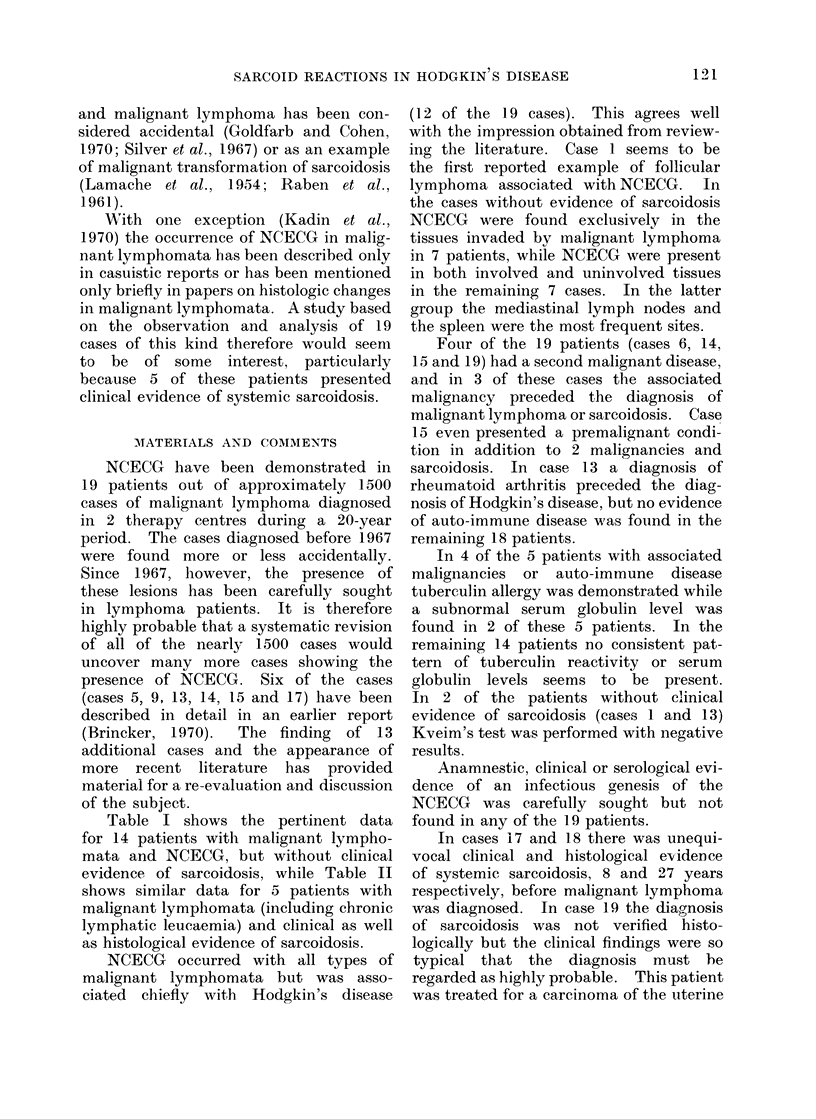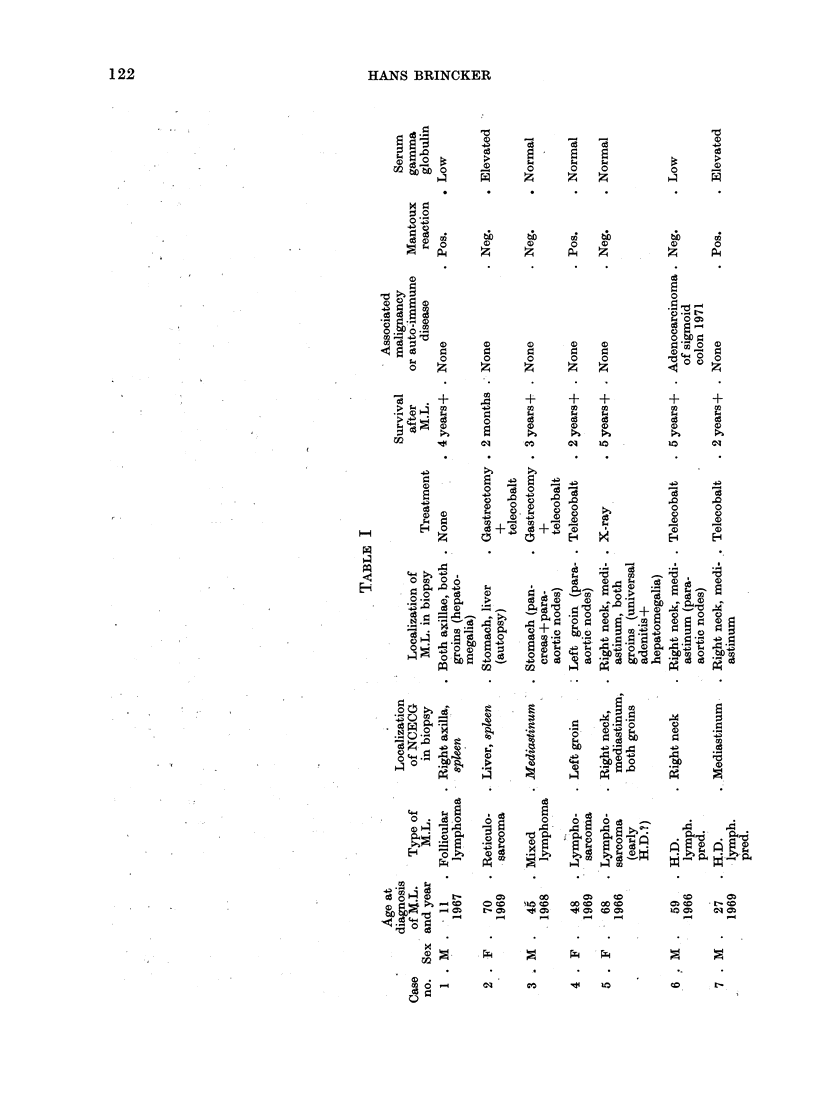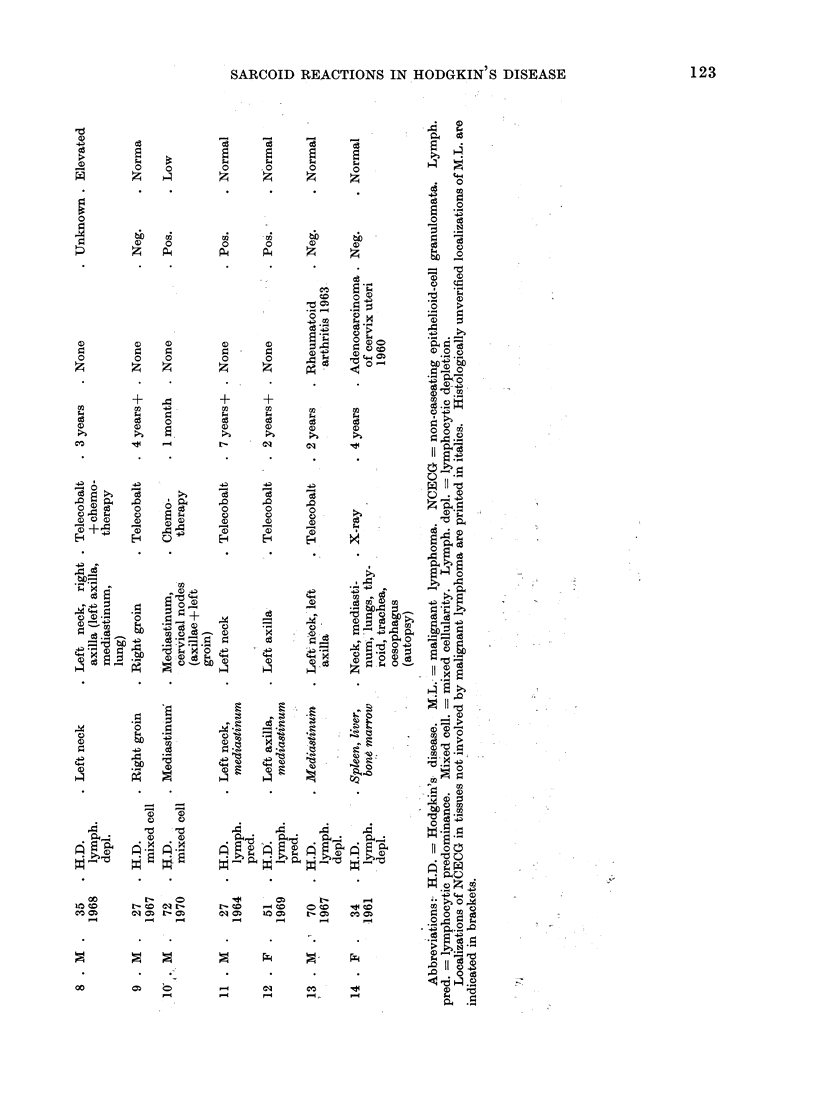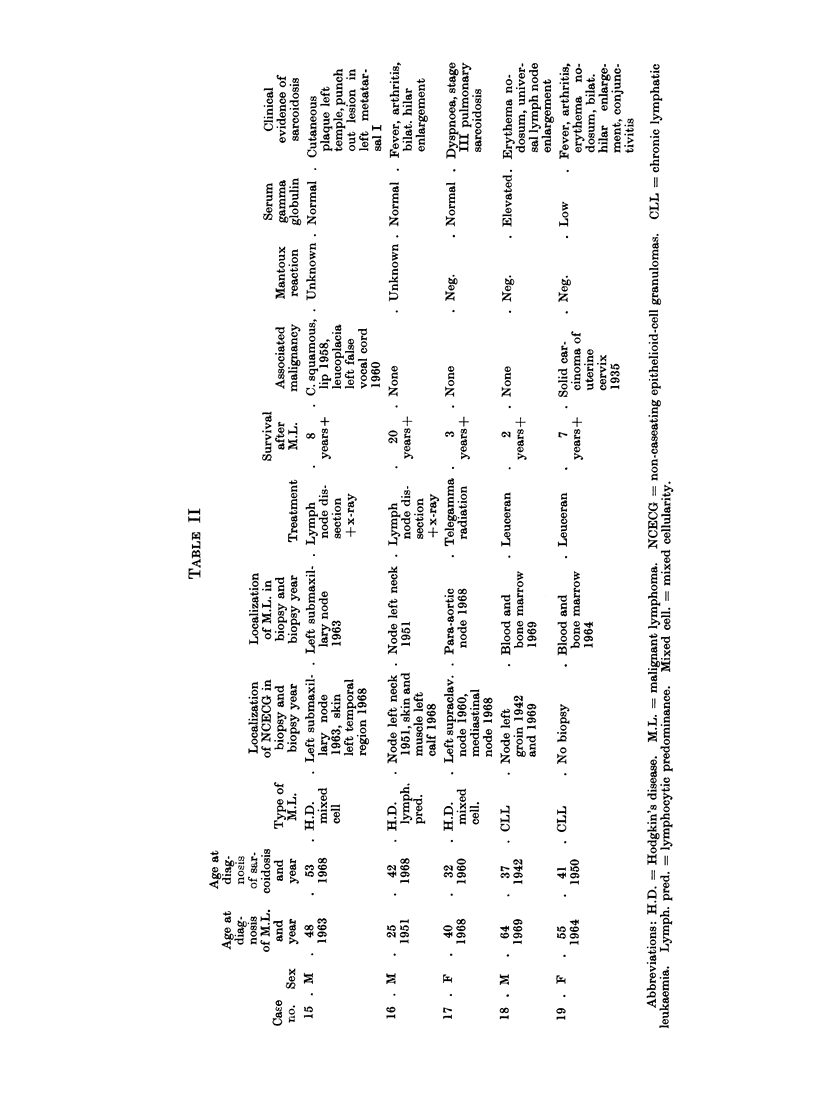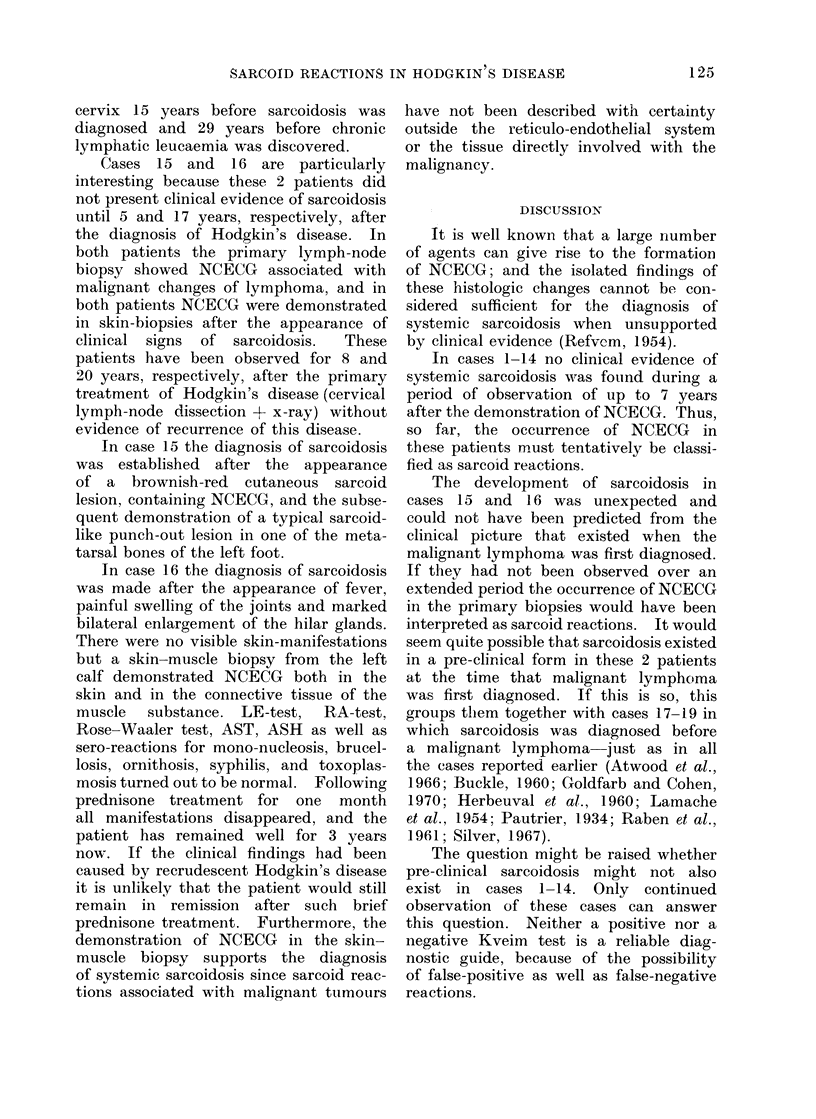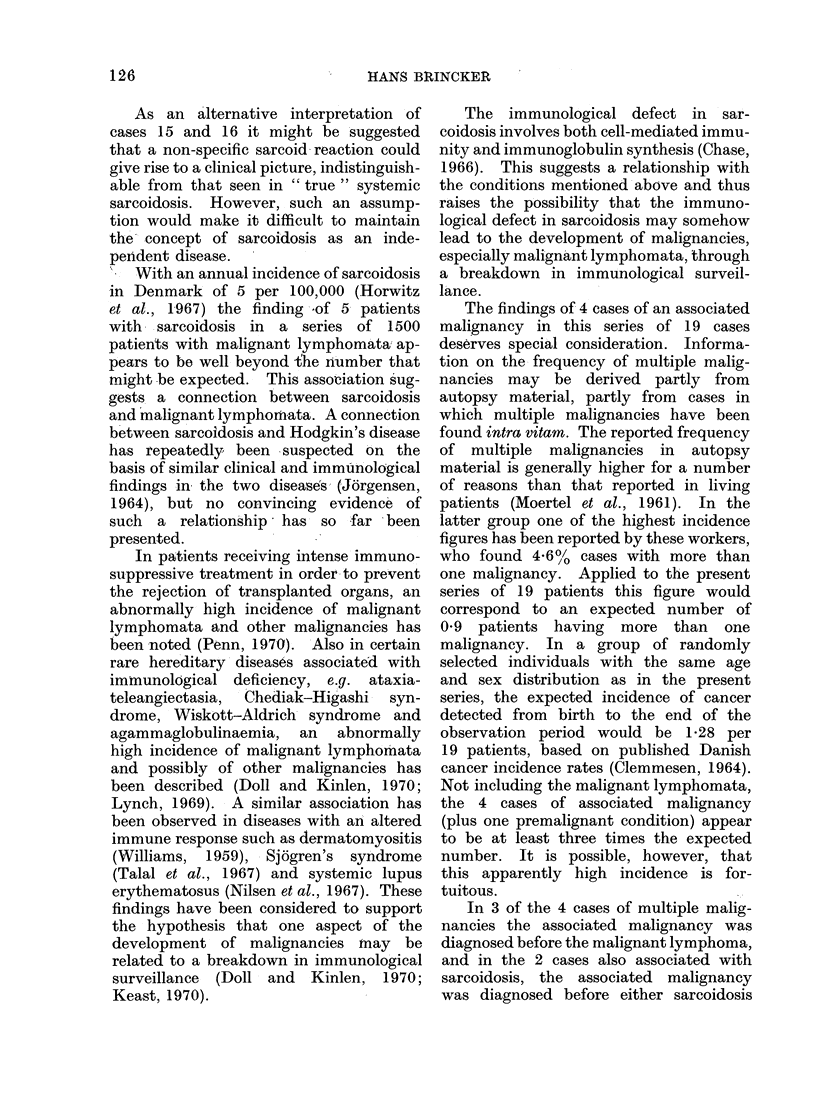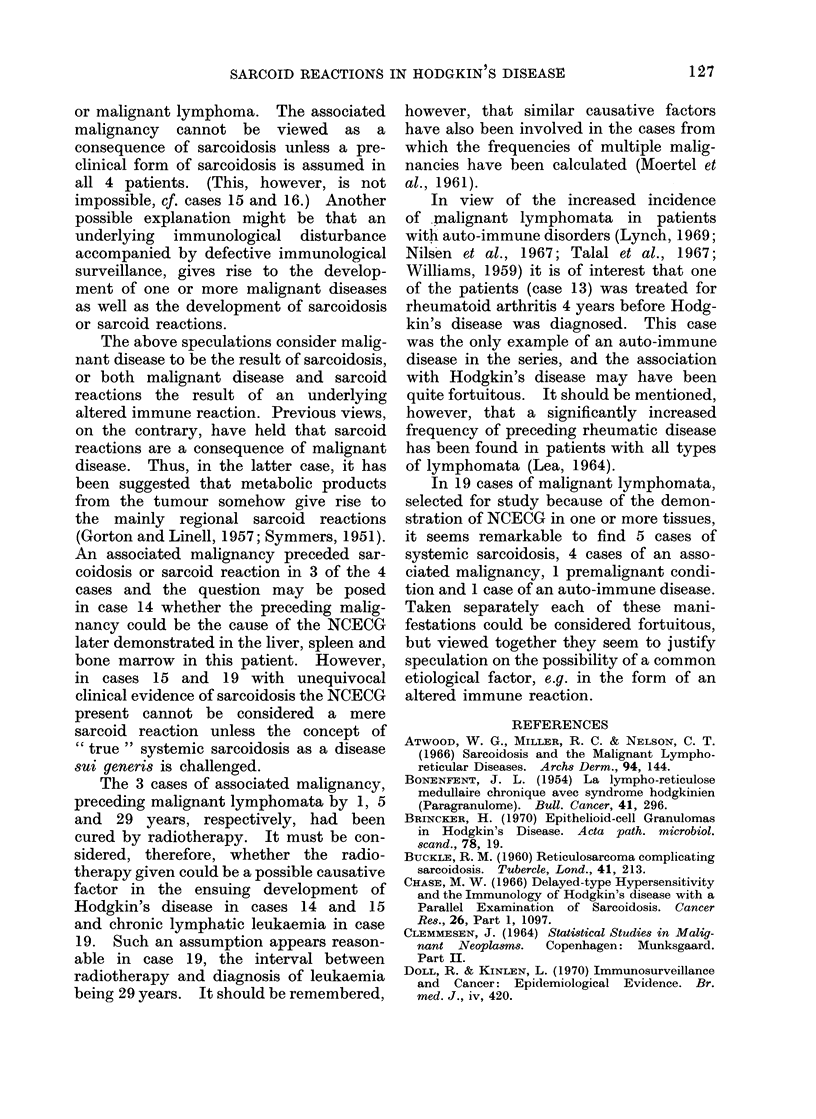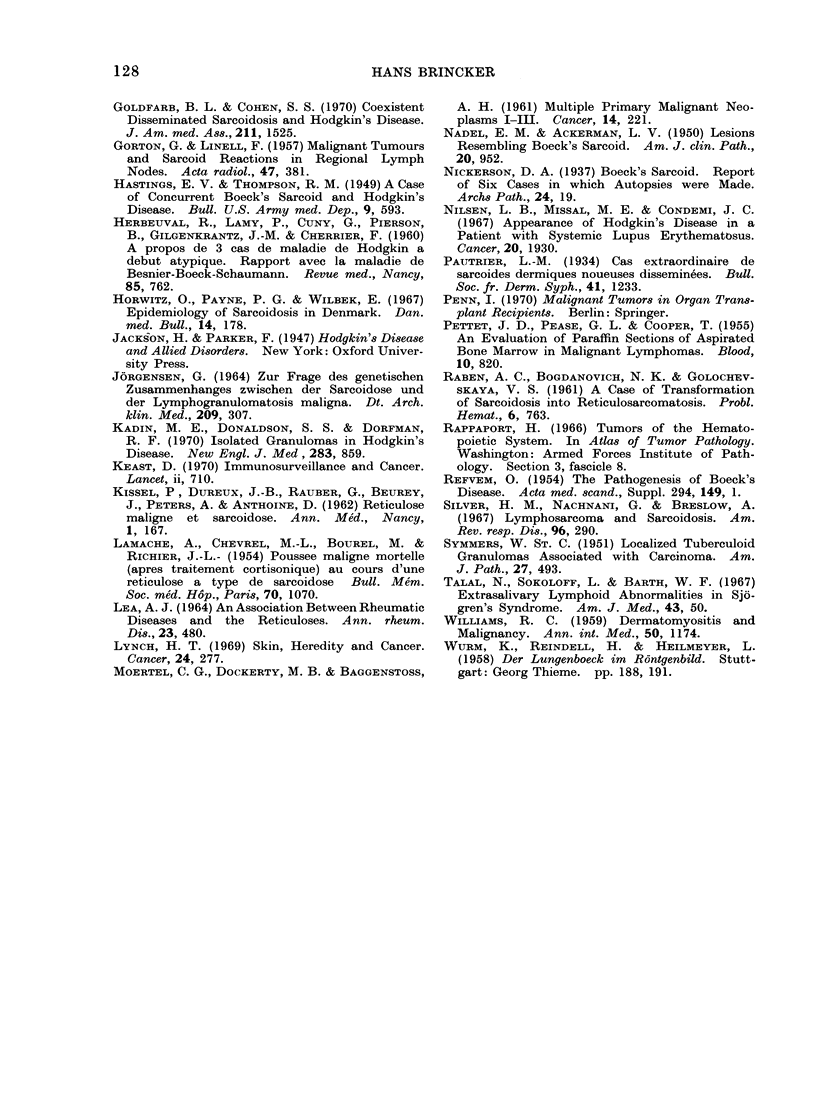# Sarcoid Reactions and Sarcoidosis in Hodgkin's Disease and Other Malignant Lymphomata

**DOI:** 10.1038/bjc.1972.18

**Published:** 1972-04

**Authors:** Hans Brincker

## Abstract

Nineteen cases of malignant lymphomata were selected for study from a group of about 1500 collected cases of malignant lymphomata because they showed histological evidence of non-infectious epithelioid-cell granulomata in one or more tissues.

In the 19 cases selected, 5 cases of systemic sarcoidosis, 4 cases of an associated malignancy, 1 pre-malignant condition, and 1 case of an auto-immune disease were found.

This remarkable association of sarcoid reactions or sarcoidosis with malignant lymphomata and associated malignancies seems to justify speculation on the possibility of a common aetiological factor, *e.g.* in the form of an altered immune reaction.


*Br. J. Cancer* (1972) **26**, 120

### SARCOID REACTIONS AND SARCOIDOSIS IN HODGKIN'S DISEASE AND OTHER MALIGNANT LYMPHOMATA

HANS BRINCKER

*From the Radium Centre, Odense Hospital and University, Odense, Denmark*

Received for publication October 1971

**Summary.—Nineteen cases of malignant lymphomata were selected for study from a group of about 1500 collected cases of malignant lymphomata because they showed histological evidence of non-infectious epithelioid-cell granulomata in one or more tissues.**

**In the 19 cases selected, 5 cases of systemic sarcoidosis, 4 cases of an associated malignancy, 1 pre-malignant condition, and 1 case of an auto-immune disease were found.**

**This remarkable association of sarcoid reactions or sarcoidosis with malignant lymphomata and associated malignancies seems to justify speculation on the possibility of a common aetiological factor, *e.g.* in the form of an altered immune reaction.**

For many years it has been known that in patients with Hodgkin's disease non-caseating epithelioid-cell granulomata (NCECG) may occur without any demonstrable infectious or other exogenous genesis (Bonenfent, 1954; Jackson and Parker, 1947; Kadin *et al.*, 1970; Nickerson, 1937; Pettet *et al.*, 1955; Rappaport, 1966). In such patients these granulomata may be found both in tissues directly involved by Hodgkin's disease as well as in uninvolved tissues; and as a rule no clinical evidence of systemic sarcoidosis is present (Goldfarb and Cohen, 1970; Hastings and Thompson, 1949; Herbeuval *et al.*, 1960; Kadin *et al.*, 1970; Wurm *et al.*, 1958). In a small number of cases, however, coexistence of " true " systemic sarcoidosis and Hodgkin's disease, has been reported, and in all these cases sarcoidosis was diagnosed first (Goldfarb and Cohen, 1970; Herbeuval *et al.*, 1960; Lamache *et al.*, 1954; Pautrier, 1934). NCECG with (Atwood *et al.*, 1966; Buckle, 1960; Raben *et al.*, 1961; Silver *et al.*, 1967) or without (Kissel *et al.*, 1962) clinical evidence of systemic sarcoidosis have also been reported in patients with other malignant lymphomata, though less frequently, and again in these cases with coexistence of sarcoidosis and malignant lymphoma the former disease has been diagnosed first.

The significance of NCECG in some patients with malignant lymphoma is still obscure and the frequency of this finding in different kinds of lymphomata is not well known. In a recent series, however, NCECG were found in 31 of 185 patients with Hodgkin's disease (Kadin *et al.*, 1970). In the cases where NCECG have been observed exclusively in the tissues invaded by Hodgkin's disease, this finding has been thought to represent an especially highly differentiated variety of the histiocytic epithelioid-cell proliferation commonly occurring in Hodgkin's disease (Bonenfent, 1954; Herbeuval *et al.*, 1960; Pautrier, 1934). The demonstration of NCECG in uninvolved organs in patients with malignant lymphomata has generated speculation on a possible underlying immunological mechanism (Atwood *et al.*, 1966; Brincker, 1970; Kadin *et al.*, 1970) or has been interpreted as sarcoid reactions (Kadin *et al.*, 1970; Kissel *et al.*, 1962) analogous to the sarcoid reactions occasionally seen in lymph-nodes draining an area involved with carcinoma (Gorton and Linell, 1957; Nadel and Ackerman, 1950; Nickerson, 1937; Symmers, 1951). Finally coexistence of systemic sarcoidosis and malignant lymphoma has been considered accidental (Goldfarb and Cohen, 1970; Silver *et al.*, 1967) or as an example of malignant transformation of sarcoidosis (Lamache *et al.*, 1954; Raben *et al.*, 1961).

With one exception (Kadin *et al.*, 1970) the occurrence of NCECG in malignant lymphomata has been described only in casuistic reports or has been mentioned only briefly in papers on histologic changes in malignant lymphomata. A study based on the observation and analysis of 19 cases of this kind therefore would seem to be of some interest, particularly because 5 of these patients presented clinical evidence of systemic sarcoidosis.

### MATERIALS AND COMMENTS

NCECG have been demonstrated in 19 patients out of approximately 1500 cases of malignant lymphoma diagnosed in 2 therapy centres during a 20-year period. The cases diagnosed before 1967 were found more or less accidentally. Since 1967, however, the presence of these lesions has been carefully sought in lymphoma patients. It is therefore highly probable that a systematic revision of all of the nearly 1500 cases would uncover many more cases showing the presence of NCECG. Six of the cases (cases 5, 9, 13, 14, 15 and 17) have been described in detail in an earlier report (Brincker, 1970). The finding of 13 additional cases and the appearance of more recent literature has provided material for a re-evaluation and discussion of the subject.

Table I shows the pertinent data for 14 patients with malignant lymphomata and NCECG, but without clinical evidence of sarcoidosis, while Table II shows similar data for 5 patients with malignant lymphomata (including chronic lymphatic leucaemia) and clinical as well as histological evidence of sarcoidosis.

NCECG occurred with all types of malignant lymphomata but was associated chiefly with Hodgkin's disease (12 of the 19 cases). This agrees well with the impression obtained from reviewing the literature. Case 1 seems to be the first reported example of follicular lymphoma associated with NCECG. In the cases without evidence of sarcoidosis NCECG were found exclusively in the tissues invaded by malignant lymphoma in 7 patients, while NCECG were present in both involved and uninvolved tissues in the remaining 7 cases. In the latter group the mediastinal lymph nodes and the spleen were the most frequent sites.

Four of the 19 patients (cases 6, 14, 15 and 19) had a second malignant disease, and in 3 of these cases the associated malignancy preceded the diagnosis of malignant lymphoma or sarcoidosis. Case 15 even presented a premalignant condition in addition to 2 malignancies and sarcoidosis. In case 13 a diagnosis of rheumatoid arthritis preceded the diagnosis of Hodgkin's disease, but no evidence of auto-immune disease was found in the remaining 18 patients.

In 4 of the 5 patients with associated malignancies or auto-immune disease tuberculin allergy was demonstrated while a subnormal serum globulin level was found in 2 of these 5 patients. In the remaining 14 patients no consistent pattern of tuberculin reactivity or serum globulin levels seems to be present. In 2 of the patients without clinical evidence of sarcoidosis (cases 1 and 13) Kveim's test was performed with negative results.

Anamnestic, clinical or serological evidence of an infectious genesis of the NCECG was carefully sought but not found in any of the 19 patients.

In cases 17 and 18 there was unequivocal clinical and histological evidence of systemic sarcoidosis, 8 and 27 years respectively, before malignant lymphoma was diagnosed. In case 19 the diagnosis of sarcoidosis was not verified histologically but the clinical findings were so typical that the diagnosis must be regarded as highly probable. This patient was treated for a carcinoma of the uterine

TABLE I

| Case no. | Sex | Age at diagnosis of M.L. and year | Type of M.L. | Localization of NCECG in biopsy | Localization of M.L. in biopsy | Treatment | Survival after M.L. | Associated malignancy or auto-immune disease | Mantoux reaction | Serum gamma globulin |
|---|---|---|---|---|---|---|---|---|---|---|
| 1 | M | 11 1967 | Follicular lymphoma | Right axilla, *spleen* | Both axillae, both groins (hepatomegalia) | None | 4 years+ | None | Pos. | Low |
| 2 | F | 70 1969 | Reticulo-sarcoma | Liver, *spleen* | Stomach, liver (autopsy) | Gastrectomy + telecobalt | 2 months | None | Neg. | Elevated |
| 3 | M | 45 1968 | Mixed lymphoma | *Mediastinum* | Stomach (pancreas+para-aortic nodes) | Gastrectomy + telecobalt | 3 years+ | None | Neg. | Normal |
| 4 | F | 48 1969 | Lympho-sarcoma | Left groin | Left groin (para-aortic nodes) | Telecobalt | 2 years+ | None | Pos. | Normal |
| 5 | F | 68 1966 | Lympho-sarcoma (early H.D.?) | Right neck, mediastinum, both groins | Right neck, mediastinum, both groins (universal adenitis+hepatomegalia) | X-ray | 5 years+ | None | Neg. | Normal |
| 6 | M | 59 1966 | H.D. lymph. pred. | Right neck | Right neck, mediastinum (para-aortic nodes) | Telecobalt | 5 years+ | Adenocarcinoma of sigmoid colon 1971 | Neg. | Low |
| 7 | M | 27 1969 | H.D. lymph. pred. | Mediastinum | Right neck, mediastinum | Telecobalt | 2 years+ | None | Pos. | Elevated |

| | | | | | | | | | | |
|---|---|---|---|---|---|---|---|---|---|---|
| 8 | M | 35 1968 | H.D. lymph. depl. | Left neck | Left neck, right axilla (left axilla, mediastinum, lung) | Telecobalt +chemo-therapy | 3 years | None | Unknown | Elevated |
| 9 | M | 27 1967 | H.D. mixed cell | Right groin | Right groin | Telecobalt | 4 years+ | None | Neg. | Norma |
| 10 | M | 72 1970 | H.D. mixed cell | Mediastinum | Mediastinum, cervical nodes (axillae+left groin) | Chemo-therapy | 1 month | None | Pos. | Low |
| 11 | M | 27 1964 | H.D. lymph. pred. | Left neck, *mediastinum* | Left neck | Telecobalt | 7 years+ | None | Pos. | Normal |
| 12 | F | 51 1969 | H.D. lymph. pred. | Left axilla, *mediastinum* | Left axilla | Telecobalt | 2 years+ | None | Pos. | Normal |
| 13 | M | 70 1967 | H.D. lymph. depl. | *Mediastinum* | Left neck, left axilla | Telecobalt | 2 years | Rheumatoid arthritis 1963 | Neg. | Normal |
| 14 | F | 34 1961 | H.D. lymph. depl. | *Spleen, liver, bone marrow* | Neck, mediastinum, lungs, thyroid, trachea, oesophagus (autopsy) | X-ray | 4 years | Adenocarcinoma of cervix uteri 1960 | Neg. | Normal |

Abbreviations: H.D. = Hodgkin's disease. M.L. = malignant lymphoma. NCECG = non-caseating epithelioid-cell granulomata. Lymph. pred. = lymphocytic predominance. Mixed cell. = mixed cellularity. Lymph. depl. = lymphocytic depletion.

Localizations of NCECG in tissues not involved by malignant lymphoma are printed in italics. Histologically unverified localizations of M.L. are indicated in brackets.

TABLE II

| Case no. | Sex | Age at diagnosis of M.L. and year | Age at diagnosis of sarcoidosis and year | Type of M.L. | Localization of NCECG in biopsy and biopsy year | Localization of M.L. in biopsy and biopsy year | Treatment | Survival after M.L. | Associated malignancy | Mantoux reaction | Serum gamma globulin | Clinical evidence of sarcoidosis |
|---|---|---|---|---|---|---|---|---|---|---|---|---|
| 15 | M | 48 1963 | 53 1968 | H.D. mixed cell | Left submaxillary node 1963, skin left temporal region 1968 | Left submaxillary node 1963 | Lymph node dissection +x-ray | 8 years+ | C. squamous, lip 1958, leucoplacia left false vocal cord 1960 | Unknown | Normal | Cutaneous plaque left temple, punch out lesion in left metatarsal I |
| 16 | M | 25 1951 | 42 1968 | H.D. lymph. pred. | Node left neck 1951, skin and muscle left calf 1968 | Node left neck 1951 | Lymph node dissection +x-ray | 20 years+ | None | Unknown | Normal | Fever, arthritis, bilat. hilar enlargement |
| 17 | F | 40 1968 | 32 1960 | H.D. mixed cell. | Left supraclav. node 1960, mediastinal node 1968 | Para-aortic node 1968 | Telegamma radiation | 3 years+ | None | Neg. | Normal | Dyspnoea, stage III pulmonary sarcoidosis |
| 18 | M | 64 1969 | 37 1942 | CLL | Node left groin 1942 and 1969 | Blood and bone marrow 1969 | Leuceran | 2 years+ | None | Neg. | Elevated | Erythema nodosum, universal lymph node enlargement |
| 19 | F | 55 1964 | 41 1950 | CLL | No biopsy | Blood and bone marrow 1964 | Leuceran | 7 years+ | Solid carcinoma of uterine cervix 1935 | Neg. | Low | Fever, arthritis, erythema nodosum, bilat. hilar enlargement, conjunctivitis |

Abbreviations: H.D. = Hodgkin's disease. M.L. = malignant lymphoma. NCECG = non-caseating epithelioid-cell granulomas. CLL = chronic lymphatic leukaemia. Lymph. pred. = lymphocytic predominance. Mixed cell. = mixed cellularity.

cervix 15 years before sarcoidosis was diagnosed and 29 years before chronic lymphatic leucaemia was discovered.

Cases 15 and 16 are particularly interesting because these 2 patients did not present clinical evidence of sarcoidosis until 5 and 17 years, respectively, after the diagnosis of Hodgkin's disease. In both patients the primary lymph-node biopsy showed NCECG associated with malignant changes of lymphoma, and in both patients NCECG were demonstrated in skin-biopsies after the appearance of clinical signs of sarcoidosis. These patients have been observed for 8 and 20 years, respectively, after the primary treatment of Hodgkin's disease (cervical lymph-node dissection + x-ray) without evidence of recurrence of this disease.

In case 15 the diagnosis of sarcoidosis was established after the appearance of a brownish-red cutaneous sarcoid lesion, containing NCECG, and the subsequent demonstration of a typical sarcoid-like punch-out lesion in one of the metatarsal bones of the left foot.

In case 16 the diagnosis of sarcoidosis was made after the appearance of fever, painful swelling of the joints and marked bilateral enlargement of the hilar glands. There were no visible skin-manifestations but a skin–muscle biopsy from the left calf demonstrated NCECG both in the skin and in the connective tissue of the muscle substance. LE-test, RA-test, Rose–Waaler test, AST, ASH as well as sero-reactions for mono-nucleosis, brucellosis, ornithosis, syphilis, and toxoplasmosis turned out to be normal. Following prednisone treatment for one month all manifestations disappeared, and the patient has remained well for 3 years now. If the clinical findings had been caused by recrudescent Hodgkin's disease it is unlikely that the patient would still remain in remission after such brief prednisone treatment. Furthermore, the demonstration of NCECG in the skin–muscle biopsy supports the diagnosis of systemic sarcoidosis since sarcoid reactions associated with malignant tumours have not been described with certainty outside the reticulo-endothelial system or the tissue directly involved with the malignancy.

### DISCUSSION

It is well known that a large number of agents can give rise to the formation of NCECG; and the isolated findings of these histologic changes cannot be considered sufficient for the diagnosis of systemic sarcoidosis when unsupported by clinical evidence (Refvem, 1954).

In cases 1–14 no clinical evidence of systemic sarcoidosis was found during a period of observation of up to 7 years after the demonstration of NCECG. Thus, so far, the occurrence of NCECG in these patients must tentatively be classified as sarcoid reactions.

The development of sarcoidosis in cases 15 and 16 was unexpected and could not have been predicted from the clinical picture that existed when the malignant lymphoma was first diagnosed. If they had not been observed over an extended period the occurrence of NCECG in the primary biopsies would have been interpreted as sarcoid reactions. It would seem quite possible that sarcoidosis existed in a pre-clinical form in these 2 patients at the time that malignant lymphoma was first diagnosed. If this is so, this groups them together with cases 17–19 in which sarcoidosis was diagnosed before a malignant lymphoma—just as in all the cases reported earlier (Atwood *et al.*, 1966; Buckle, 1960; Goldfarb and Cohen, 1970; Herbeuval *et al.*, 1960; Lamache *et al.*, 1954; Pautrier, 1934; Raben *et al.*, 1961; Silver, 1967).

The question might be raised whether pre-clinical sarcoidosis might not also exist in cases 1–14. Only continued observation of these cases can answer this question. Neither a positive nor a negative Kveim test is a reliable diagnostic guide, because of the possibility of false-positive as well as false-negative reactions.

As an alternative interpretation of cases 15 and 16 it might be suggested that a non-specific sarcoid reaction could give rise to a clinical picture, indistinguishable from that seen in " true " systemic sarcoidosis. However, such an assumption would make it difficult to maintain the concept of sarcoidosis as an independent disease.

With an annual incidence of sarcoidosis in Denmark of 5 per 100,000 (Horwitz *et al.*, 1967) the finding of 5 patients with sarcoidosis in a series of 1500 patients with malignant lymphomata appears to be well beyond the number that might be expected. This association suggests a connection between sarcoidosis and malignant lymphomata. A connection between sarcoidosis and Hodgkin's disease has repeatedly been suspected on the basis of similar clinical and immunological findings in the two diseases (Jörgensen, 1964), but no convincing evidence of such a relationship has so far been presented.

In patients receiving intense immunosuppressive treatment in order to prevent the rejection of transplanted organs, an abnormally high incidence of malignant lymphomata and other malignancies has been noted (Penn, 1970). Also in certain rare hereditary diseases associated with immunological deficiency, *e.g.* ataxia-teleangiectasia, Chediak–Higashi syndrome, Wiskott–Aldrich syndrome and agammaglobulinaemia, an abnormally high incidence of malignant lymphomata and possibly of other malignancies has been described (Doll and Kinlen, 1970; Lynch, 1969). A similar association has been observed in diseases with an altered immune response such as dermatomyositis (Williams, 1959), Sjögren's syndrome (Talal *et al.*, 1967) and systemic lupus erythematosus (Nilsen *et al.*, 1967). These findings have been considered to support the hypothesis that one aspect of the development of malignancies may be related to a breakdown in immunological surveillance (Doll and Kinlen, 1970; Keast, 1970).

The immunological defect in sarcoidosis involves both cell-mediated immunity and immunoglobulin synthesis (Chase, 1966). This suggests a relationship with the conditions mentioned above and thus raises the possibility that the immunological defect in sarcoidosis may somehow lead to the development of malignancies, especially malignant lymphomata, through a breakdown in immunological surveillance.

The findings of 4 cases of an associated malignancy in this series of 19 cases deserves special consideration. Information on the frequency of multiple malignancies may be derived partly from autopsy material, partly from cases in which multiple malignancies have been found *intra vitam*. The reported frequency of multiple malignancies in autopsy material is generally higher for a number of reasons than that reported in living patients (Moertel *et al.*, 1961). In the latter group one of the highest incidence figures has been reported by these workers, who found 4·6% cases with more than one malignancy. Applied to the present series of 19 patients this figure would correspond to an expected number of 0·9 patients having more than one malignancy. In a group of randomly selected individuals with the same age and sex distribution as in the present series, the expected incidence of cancer detected from birth to the end of the observation period would be 1·28 per 19 patients, based on published Danish cancer incidence rates (Clemmesen, 1964). Not including the malignant lymphomata, the 4 cases of associated malignancy (plus one premalignant condition) appear to be at least three times the expected number. It is possible, however, that this apparently high incidence is fortuitous.

In 3 of the 4 cases of multiple malignancies the associated malignancy was diagnosed before the malignant lymphoma, and in the 2 cases also associated with sarcoidosis, the associated malignancy was diagnosed before either sarcoidosis or malignant lymphoma. The associated malignancy cannot be viewed as a consequence of sarcoidosis unless a preclinical form of sarcoidosis is assumed in all 4 patients. (This, however, is not impossible, *cf.* cases 15 and 16.) Another possible explanation might be that an underlying immunological disturbance accompanied by defective immunological surveillance, gives rise to the development of one or more malignant diseases as well as the development of sarcoidosis or sarcoid reactions.

The above speculations consider malignant disease to be the result of sarcoidosis, or both malignant disease and sarcoid reactions the result of an underlying altered immune reaction. Previous views, on the contrary, have held that sarcoid reactions are a consequence of malignant disease. Thus, in the latter case, it has been suggested that metabolic products from the tumour somehow give rise to the mainly regional sarcoid reactions (Gorton and Linell, 1957; Symmers, 1951). An associated malignancy preceded sarcoidosis or sarcoid reaction in 3 of the 4 cases and the question may be posed in case 14 whether the preceding malignancy could be the cause of the NCECG later demonstrated in the liver, spleen and bone marrow in this patient. However, in cases 15 and 19 with unequivocal clinical evidence of sarcoidosis the NCECG present cannot be considered a mere sarcoid reaction unless the concept of " true " systemic sarcoidosis as a disease *sui generis* is challenged.

The 3 cases of associated malignancy, preceding malignant lymphomata by 1, 5 and 29 years, respectively, had been cured by radiotherapy. It must be considered, therefore, whether the radiotherapy given could be a possible causative factor in the ensuing development of Hodgkin's disease in cases 14 and 15 and chronic lymphatic leukaemia in case 19. Such an assumption appears reasonable in case 19, the interval between radiotherapy and diagnosis of leukaemia being 29 years. It should be remembered, however, that similar causative factors have also been involved in the cases from which the frequencies of multiple malignancies have been calculated (Moertel *et al.*, 1961).

In view of the increased incidence of malignant lymphomata in patients with auto-immune disorders (Lynch, 1969; Nilsen *et al.*, 1967; Talal *et al.*, 1967; Williams, 1959) it is of interest that one of the patients (case 13) was treated for rheumatoid arthritis 4 years before Hodgkin's disease was diagnosed. This case was the only example of an auto-immune disease in the series, and the association with Hodgkin's disease may have been quite fortuitous. It should be mentioned, however, that a significantly increased frequency of preceding rheumatic disease has been found in patients with all types of lymphomata (Lea, 1964).

In 19 cases of malignant lymphomata, selected for study because of the demonstration of NCECG in one or more tissues, it seems remarkable to find 5 cases of systemic sarcoidosis, 4 cases of an associated malignancy, 1 premalignant condition and 1 case of an auto-immune disease. Taken separately each of these manifestations could be considered fortuitous, but viewed together they seem to justify speculation on the possibility of a common etiological factor, *e.g.* in the form of an altered immune reaction.

### REFERENCES

Atwood, W. G., Miller, R. C. & Nelson, C. T. (1966) Sarcoidosis and the Malignant Lymphoreticular Diseases. *Archs Derm.*, **94**, 144.

Bonenfent, J. L. (1954) La lympho-reticulose medullaire chronique avec syndrome hodgkinien (Paragranulome). *Bull. Cancer*, **41**, 296.

Brincker, H. (1970) Epithelioid-cell Granulomas in Hodgkin's Disease. *Acta path. microbiol. scand.*, **78**, 19.

Buckle, R. M. (1960) Reticulosarcoma complicating sarcoidosis. *Tubercle, Lond.*, **41**, 213.

Chase, M. W. (1966) Delayed-type Hypersensitivity and the Immunology of Hodgkin's disease with a Parallel Examination of Sarcoidosis. *Cancer Res.*, **26**, Part 1, 1097.

Clemmesen, J. (1964) *Statistical Studies in Malignant Neoplasms.* Copenhagen: Munksgaard. Part II.

Doll, R. & Kinlen, L. (1970) Immunosurveillance and Cancer: Epidemiological Evidence. *Br. med. J.*, iv, 420.

GOLDFARB, B. L. & COHEN, S. S. (1970) Coexistent Disseminated Sarcoidosis and Hodgkin's Disease. *J. Am. med. Ass.*, **211**, 1525.

GORTON, G. & LINELL, F. (1957) Malignant Tumours and Sarcoid Reactions in Regional Lymph Nodes. *Acta radiol.*, **47**, 381.

HASTINGS, E. V. & THOMPSON, R. M. (1949) A Case of Concurrent Boeck's Sarcoid and Hodgkin's Disease. *Bull. U.S. Army med. Dep.*, **9**, 593.

HERBEUVAL, R., LAMY, P., CUNY, G., PIERSON, B., GILGENKRANTZ, J.-M. & CHERRIER, F. (1960) A propos de 3 cas de maladie de Hodgkin a debut atypique. Rapport avec la maladie de Besnier-Boeck-Schaumann. *Revue med., Nancy*, **85**, 762.

HORWITZ, O., PAYNE, P. G. & WILBEK, E. (1967) Epidemiology of Sarcoidosis in Denmark. *Dan. med. Bull.*, **14**, 178.

JACKSON, H. & PARKER, F. (1947) *Hodgkin's Disease and Allied Disorders.* New York: Oxford University Press.

JÖRGENSEN, G. (1964) Zur Frage des genetischen Zusammenhanges zwischen der Sarcoidose und der Lymphogranulomatosis maligna. *Dt. Arch. klin. Med.*, **209**, 307.

KADIN, M. E., DONALDSON, S. S. & DORFMAN, R. F. (1970) Isolated Granulomas in Hodgkin's Disease. *New Engl. J. Med* , **283**, 859.

KEAST, D. (1970) Immunosurveillance and Cancer. *Lancet*, ii, 710.

KISSEL, P , DUREUX, J.-B., RAUBER, G., BEUREY, J., PETERS, A. & ANTHOINE, D. (1962) Reticulose maligne et sarcoidose. *Ann. Méd., Nancy*, **1**, 167.

LAMACHE, A., CHEVREL, M.-L., BOUREL, M. & RICHIER, J.-L.- (1954) Poussee maligne mortelle (apres traitement cortisonique) au cours d'une reticulose a type de sarcoidose *Bull. Mém. Soc. méd. Hôp., Paris*, **70**, 1070.

LEA, A. J. (1964) An Association Between Rheumatic Diseases and the Reticuloses. *Ann. rheum. Dis.*, **23**, 480.

LYNCH, H. T. (1969) Skin, Heredity and Cancer. *Cancer*, **24**, 277.

MOERTEL, C. G., DOCKERTY, M. B. & BAGGENSTOSS, A. H. (1961) Multiple Primary Malignant Neoplasms I–III. *Cancer*, **14**, 221.

NADEL, E. M. & ACKERMAN, L. V. (1950) Lesions Resembling Boeck's Sarcoid. *Am. J. clin. Path.*, **20**, 952.

NICKERSON, D. A. (1937) Boeck's Sarcoid. Report of Six Cases in which Autopsies were Made. *Archs Path.*, **24**, 19.

NILSEN, L. B., MISSAL, M. E. & CONDEMI, J. C. (1967) Appearance of Hodgkin's Disease in a Patient with Systemic Lupus Erythematosus. *Cancer*, **20**, 1930.

PAUTRIER, L.-M. (1934) Cas extraordinaire de sarcoides dermiques noueuses disseminées. *Bull. Soc. fr. Derm. Syph.*, **41**, 1233.

PENN, I. (1970) *Malignant Tumors in Organ Transplant Recipients.* Berlin: Springer.

PETTET, J. D., PEASE, G. L. & COOPER, T. (1955) An Evaluation of Paraffin Sections of Aspirated Bone Marrow in Malignant Lymphomas. *Blood*, **10**, 820.

RABEN, A. C., BOGDANOVICH, N. K. & GOLOCHEVSKAYA, V. S. (1961) A Case of Transformation of Sarcoidosis into Reticulosarcomatosis. *Probl. Hemat.*, **6**, 763.

RAPPAPORT, H. (1966) Tumors of the Hematopoietic System. In *Atlas of Tumor Pathology.* Washington: Armed Forces Institute of Pathology. Section 3, fascicle 8.

REFVEM, O. (1954) The Pathogenesis of Boeck's Disease. *Acta med. scand.*, Suppl. 294, **149**, 1.

SILVER, H. M., NACHNANI, G. & BRESLOW, A. (1967) Lymphosarcoma and Sarcoidosis. *Am. Rev. resp. Dis.*, **96**, 290.

SYMMERS, W. ST. C. (1951) Localized Tuberculoid Granulomas Associated with Carcinoma. *Am. J. Path.*, **27**, 493.

TALAL, N., SOKOLOFF, L. & BARTH, W. F. (1967) Extrasalivary Lymphoid Abnormalities in Sjögren's Syndrome. *Am. J. Med.*, **43**, 50.

WILLIAMS, R. C. (1959) Dermatomyositis and Malignancy. *Ann. int. Med.*, **50**, 1174.

WURM, K., REINDELL, H. & HEILMEYER, L. (1958) *Der Lungenboeck im Röntgenbild.* Stuttgart: Georg Thieme. pp. 188, 191.